# Beyond the Benchmarks: Toward Human-Like Lexical Representations

**DOI:** 10.3389/frai.2022.796741

**Published:** 2022-05-24

**Authors:** Suzanne Stevenson, Paola Merlo

**Affiliations:** ^1^Department of Computer Science, University of Toronto, Toronto, ON, Canada; ^2^Linguistics Department, University of Geneva, Geneva, Switzerland

**Keywords:** computational linguistics, natural language processing, lexical semantics, lexicon structure, human lexical representations, cross-linguistic generalization

## Abstract

To process language in a way that is compatible with human expectations in a communicative interaction, we need computational representations of lexical properties that form the basis of human knowledge of words. In this article, we concentrate on word-level semantics. We discuss key concepts and issues that underlie the scientific understanding of the human lexicon: its richly structured semantic representations, their ready and continual adaptability, and their grounding in crosslinguistically valid conceptualization. We assess the state of the art in natural language processing (NLP) in achieving these identified properties, and suggest ways in which the language sciences can inspire new approaches to their computational instantiation.

## 1. Introduction

The field of computational linguistics (CL) has exploded recently—especially the work characterized as “NLP,” which has become almost synonymous with “machine learning approaches applied to large text datasets.” The practical successes have been rampant (e.g., Collobert and Weston, 2008; Mikolov et al., 2013a; Bahdanau et al., 2015; Vaswani et al., 2017; Devlin et al., 2018). But the progress on task-oriented measures and benchmark performance has come at a price. CL as a field has long benefited from bringing together insights from theoretical linguistics, psycholinguistics, and other of the language sciences, to inform computational methods for automatically processing language. This inherently interdisciplinary approach has over time helped to ensure that computational systems are grounded in firm scientific understanding of the nature of human language. Periodically, however, the success of a particular computational approach has threatened this interdisciplinarity by seeming to obviate the need for drawing on other disciplines; this phenomenon has perhaps been most famously captured by the saying from Fred Jelinek, “Whenever I fire a linguist our system performance improves”.^1^

We are today in NLP seeing a similar emphasis on performance and an associated focus on a particular class of algorithms.^2^ This exclusionary focus is unfortunate, as it has meant that CL has weakened its crucial connections to the other language sciences, and thereby lost some of the underpinnings and guidance that comes from a comprehensive scientific understanding of language as an essential and uniquely human ability.^3^ Although recent NLP work can achieve performance on benchmarks that was unheard of only a short time ago, one might wonder how much such research is actually furthering progress on the overarching goal of matching broad human abilities in linguistic communication.

In this article, we consider this issue in the context of research on lexical semantics. We adopt this focus for two reasons. First, lexical items—or words^4^—are the locus of fundamental semantics, as well as of combinatorial properties that underlie their composition into the larger units of meaning used in communication. As such, words are a basic building block of language, and adequately capturing lexical semantics is critical to computational systems for processing language. Second, words generally—and lexical semantics in particular—have received much attention in recent NLP, and are the focus of many of the reported successes noted above.

Specifically, our aim here is to examine essential desiderata for computational approaches to lexical semantics. In order to process language in a way that is compatible with human expectations in a communicative interaction, we need computational representations of lexical properties that adequately capture human knowledge of words. In this context, we discuss the concepts and issues that underlie the scientific understanding of the human lexicon and key defining properties (Section 2); assess the state of the art in NLP in achieving the identified properties (Section 3); and suggest ways in which the language sciences can inspire new approaches to their computational instantiation (Section 4).

## 2. The Human Lexicon

The human capacity for language is founded in very powerful cognitive mechanisms that underlie general intelligence: the ability to (multiply) categorize stimuli into richly structured representations, and to continually learn and readily adapt to novel stimuli (e.g., Langacker, 1987; Croft and Cruse, 2004; Goldberg, 2006). Moreover, language is grounded in universal human experience, such that these categorization and generalization mechanisms operate over a level of universal (crosslinguistically valid) conceptual grounding (e.g., Berlin and Kay, 1969; Bowerman and Choi, 2001; Levinson et al., 2003; Regier et al., 2007; Majid et al., 2008; Gentner and Bowerman, 2009). We briefly discuss the implications of each of these three properties for the human lexicon.

First, human lexical representations and the lexicon itself exhibit a rich semantic structure, encoding a multitude of semantic relations among words. In addition to semantic similarity and semantic relatedness,^5^ people are sensitive to taxonomic relations, part-whole relations, entailment, subsumption, hyponymy, and many others, which organize the meaning of words and their relation to each other in a multiply connected structure (e.g., Collins and Loftus, 1975; Pustejovsky, 1995; Miller, 1998; Hale and Keyser, 2002; Jones et al., 2015). In addition to the structured relations among them, words also have rich internal semantic structure (e.g., Cruse, 1986; Pustejovsky, 1995; Croft and Cruse, 2004). Moreover, the commonalities along various semantic dimensions can form the basis for classes of words that have shared linguistic behavior, thus serving as a critically important means for organizing further grammatical knowledge (e.g., Levin, 1993; Croft, 1994; Baker, 2003). Lexical representation is further complicated by lexical ambiguity: Most words have multiple meanings (Bréal, 1897), with a high degree of variability in the extent and manner in which those meanings are related (e.g., Nunberg, 1979; Bartsch, 1984; Williams, 1992; Geeraerts, 1993; Tuggy, 1993), which people are sensitive to (e.g., Rodd et al., 2002; Klepousniotou et al., 2008; Armstrong and Plaut, 2016). Words are thus linked to each other by elaborate networks of semantic relations that are crucial to their felicitous use and combination.

Second, human lexical representations are malleable: in addition to being multiply ambiguous, they are readily amenable to meaning shifts in context, and frequently undergo semantic change, taking on new senses. This online adaptability is the key to successful interaction. People not only easily access different aspects of meanings in different contexts, they construct nuanced interpretations in conjunction with conversational partners (e.g., Clark and Clark, 1979; Langacker, 1987; Brennan and Clark, 1996; Cruse, 2000; Kintsch, 2001; Croft and Cruse, 2004; Zawada, 2006). Such representations are not always fleeting: linguistic creativity entails that people frequently generate new usages of words and shifts in meaning, and interlocutors adjust their lexical knowledge in response to such novel usages (e.g., Langacker, 1987; Croft and Cruse, 2004; Goldberg, 2006). While it has long been recognized that children have inductive biases to help them learn from small amounts of data (e.g., Clark, 1987; Markman, 1987; Samuelson and Smith, 1999), the dynamic nature of the human lexicon means that adults also are continually adapting their lexical representations.

Third, and finally, lexical semantic knowledge is built on universal principles that are grounded in fundamental human perceptual and conceptual experiences that hold across languages (e.g., Goddard and Wierzbicka, 1994; Haspelmath, 1997; Regier et al., 2007; Majid et al., 2008; Majid and Van Staden, 2015; Kemp et al., 2018). The result is that languages show constrained variation in their lexical semantic systems. For example, languages vary widely in the precise lexical divisions they adopt in a domain (such as how to carve up the continuous color spectrum into basic color terms), differentially making a trade-off between expressivity of the terms and efficiency in their lexicons (e.g., Kemp et al., 2018; Zaslavsky et al., 2018). However, considerations of “cognitive naturalness” of lexical categories greatly constrain the observed variation across languages, such that human lexicons follow common organizational principles (e.g., Berlin and Kay, 1969; Levinson et al., 2003; Gentner and Bowerman, 2009; Xu et al., 2020). Moreover, people benefit (or suffer!) from “transfer effects” in learning a new language, or in lexical access in the context of a multilingual lexicon (e.g., Van Hell and de Groot, 1998; Degani et al., 2011). Thus, the universality of the cognitive/conceptual basis of language leads to predictions about expected crosslinguistic commonalities and areas of difference.

## 3. Assessing The Lexical Representations in NLP

The identified properties of the human lexicon—richly structured representations, ready and continual adaptability, and universality—have been differentially highlighted at different stages of development in NLP, but have rarely been addressed comprehensively. In the first subsection below, we briefly outline some of the relevant history of computational lexical semantics, presenting the progression of ideas with reference to these key properties. In the second subsection, we discuss ways in which the current state-of-the-art in lexical semantic representation continues to fall short of the identified properties of the lexicon that support successful human communication.

### 3.1. From Early Structured to Distributional to Neural Approaches

Achieving broad coverage lexical knowledge has long been recognized as a critical step to achieving language processing at scale (i.e., beyond narrow domains or circumscribed tasks). Early approaches to large-scale lexical resources focused on highly structured lexical representations, as in, for example, WordNet (Beckwith et al., 1991; Fellbaum, 1998), FrameNet (Baker et al., 1998; Fillmore and Atkins, 1998), VerbNet (Levin, 1993; Kipper, 2005), and PropBank (Palmer et al., 2005). The structure of such lexicons is not only a practical organizational technique: crucially, lexical items derive their nuanced semantics in part through the elaboration of multiple semantic and/or syntactic relations among them. For example, Wordnet organizes words into *synsets* that group roughly synonymous words, and then links these synsets with hypernym/hyponym links (among other semantic relations) to indicate a basic taxonomic structure over meanings. Due to ambiguity, words can appear in multiple synsets, leading to a complex network structure. For example, one of the synsets of *newspaper* is {newspaper, paper} whose hypernym is {press, public press}, while another synset is {newspaper, newsprint}, whose hypernym is {paper}, where the word *paper* in different senses is both a synonym and a hypernym of *newspaper*. These resources thus capture rich semantic structure that has supported a range of applications, such as word sense disambiguation (e.g., Patwardhan et al., 2003), semantic parsing (e.g., Das et al., 2014), and question-answering (e.g., Clark et al., 2018). However, while these resources have been very successfully deployed for key tasks in NLP, they are difficult to adapt dynamically, and require considerable manual effort to transfer to other languages, because of the necessity for elaborating the multiple senses and/or semantic relations for each word (e.g., Vossen, 1998; Burchardt et al., 2009). In short, the very richness of their structure makes it resource-intensive to extend them within or across languages.

In response to these shortcomings, automatic lexical acquisition was identified as key to further progress in CL (e.g., Ellison, 1997; Baldwin et al., 2005; Armstrong et al., 2010). Computational work in lexical semantics in the 1990s and 2000s had two prominent strands: learning of the structured relations among words (a key source of the power of the above resources), as well as learning the meaning of individual words. In both cases, the focus on learning from data was intended to address both the need for adaptability and the desire for crosslinguistic breadth and validity.

The first strand of work in data-driven lexical acquisition concentrated on structured lexical representations. For example, much work aimed to learn various semantic relations among words, such as hyponymy, synonymy, part-whole, etc. (e.g., Hearst, 1992; Riloff, 1996; Girju et al., 2006). For example, the simple but highly effective technique of “Hearst patterns” used common phrases to automatically infer taxonomic relations among words; e.g., “HYPERNYM*, such as* HYPONYM” (*fruit, such as apples and bananas*) or “HYPONYM *and other* HYPERNYM” (*apples and other fruit*). Another important focus was on automatically acquiring the rich information about predicates (such as argument structure and verb or adjective classes). These methods used statistics over the syntactic patterns of predicates to automatically classify them into known semantic classes, or even to discover such classes, in order to generalize known combinatory properties of lexical items to novel or previously unseen words (Merlo and Stevenson, 2001, 2005; Stevenson and Joanis, 2003; Boleda et al., 2004; Korhonen and Briscoe, 2004; Schulte im Walde, 2006; Li and Brew, 2008; Sun and Korhonen, 2009). In addition to achieving adaptability within a language, some research was driven by the goal of crosslinguistic adaptability as well. For example, some approaches exploited crosslinguistic similarities to extend methods developed for English to new languages (Merlo et al., 2002; Padó and Lapata, 2005; Snyder and Barzilay, 2008; Burchardt et al., 2009; Samardžić and Merlo, 2010). Other work leveraged multi-lingual resources further, by using knowledge of crosslinguistic variation as a way to improve results *within* a language. For example, Tsang et al. (2002) exploited bilingual corpus data to learn a semantic distinction in English that is not morphologically marked in English, but is so marked in Chinese. Despite these various advances in automatic lexical acquisition, a challenge remained for structured lexical approaches: These methods relied on identifying surface correlates of the deeper semantic properties to be learned, which often had to be done manually.

The approaches above were using distributional patterns to learn a set of semantic relations or an assignment into a (typically pre-conceived) structured representation. At the same time, researchers were increasingly considering distributional cues as capable of comprising the semantic representation itself. A wealth of work on distributional semantic models (DSMs) was inspired by early views in linguistics and philosophy that meaning is determined by use in context (Wittgenstein, 1953; Harris, 1954; Firth, 1957), and by computational cognitive modeling approaches to capturing meaning based on word contextual associations (e.g., Lund and Burgess, 1996; Landauer and Dumais, 1997). In contrast to the structured lexical approaches described above, the distributional hypothesis promised a data-driven representation of semantics that would avoid both the manual work and the need for explicit assumptions about semantics that may not generalize across domains, genres, and languages. Moreover, such representations had the potential to capture the various senses of a lexical item, which could be disambiguated in composition with co-occurring words (e.g., Landauer and Dumais, 1997; Kintsch, 2001; Erk and Padó, 2008; Mitchell and Lapata, 2008; Van de Cruys et al., 2011). Many types of DSM approaches have been explored in CL, considering various context sizes (e.g., number of words, or neighboring words vs. documents), contextual relations (e.g., word co-occurrence vs. dependency relations), and statistical measures of word–context association (Schütze, 1994; Padó and Lapata, 2007; Erk and Padó, 2008; Mitchell and Lapata, 2010). DSMs have generally yielded semantic representations that perform well on semantic similarity benchmarks and in a range of downstream NLP tasks (Schütze, 1994; Landauer and Dumais, 1997; Baroni and Lenci, 2010). By the mid 2000s, DSMs had become a prominent means of lexical semantic representation in CL (e.g., Lenci, 2008).

More recently, the increased power of statistical methods and neural network approaches have enabled DSMs to exploit the promise of the distributional hypothesis to a high degree (Collobert and Weston, 2007; Mikolov et al., 2013c; Pennington et al., 2014; Pereira et al., 2016), and the techniques have been successfully applied across many languages (Bojanowski et al., 2017). Moreover, recent methods have extended the basic framework to integrate with neural language models, thereby achieving adaptability of meanings in local (sentence-level) contexts for many languages (e.g., ELMo, BERT, mBERT; Devlin et al., 2018; Peters et al., 2018; Wu and Dredze, 2019). The broad practical successes of neural approaches to learning word meaning and integrating lexical semantics with other NLP tasks has led to their current dominance in the field.

### 3.2. Current Limitations in Matching Human Lexical Properties

Despite their success, distributional semantic representations—“word embeddings”—are still far from capturing human-like lexical abilities, along all the dimensions of structure, adaptability, and universality. First, current word embeddings do not encode all of the rich semantic properties and relations that we know humans are sensitive to (e.g., Rubinstein et al., 2015; Boleda et al., 2017; Grand et al., 2018). For example, Rubinstein et al. (2015) found that word embeddings captured taxonomic knowledge (‘is a fruit’, ‘is an animal’) much better than they did attributive properties of word meanings (‘is yellow,’ ‘is round’). With regard to ambiguity, while evidence suggests that distributional word representations can capture multiple meanings of a word (Burgess, 2001; Kintsch, 2001; Mu et al., 2017; Arora et al., 2018; Beekhuizen et al., 2019), much remains to be explored about whether and how they might do so (Reisinger and Mooney, 2010; Li and Jurafsky, 2015; Jamieson et al., 2018). In addition, while much earlier lexical acquisition work successfully learned verb argument structures and their surface expression, experiments on context-aware embeddings have shown inconsistent performance in predicting the valid usages of verbs (e.g., Kann et al., 2019; Warstadt et al., 2019). While there is legitimate skepticism that purely text-based distributional methods can truly learn human-like meanings (e.g., Sahlgren, 2008; Bender and Koller, 2020), there is also much room for them to extend their capabilities beyond solely similarity-based semantic space.

Second, contextualized word embeddings have shown some success at exhibiting nuance of meaning in context (e.g., Choi et al., 2017; Ethayarajh, 2019; Hofmann et al., 2020). However, at least some approaches are overly sensitive to irrelevant factors (e.g., word order variation that does not change meaning), such that very close paraphrases are not assigned close embeddings (Shi et al., 2019). Further research will need to assess how well current approaches to contextualized understanding of words matches that of people. Moreover, while research on historical semantic change has thrived using historical embeddings (e.g., Hamilton et al., 2016; Lu et al., 2019), little attention has been paid to shorter-term sense change, with some caveats for using word embeddings in this task (Del Tredici et al., 2018). In addition, while there has been much focus on one-shot or few-shot learning as a means for adapting the knowledge of large-scale models (e.g., Li et al., 2006; Ritter et al., 2017; Brown et al., 2020; Schick and Schütze, 2021), recent work has discussed that “few-shot” learning is not as data-lean as it may seem (Perez et al., 2021). Thus, although their foundation in learning from data holds the promise of adaptability, the data requirements of neural approaches can limit their ability to adapt on-the-fly in the way that people can.

Finally, it is not yet clear how “universal” are the current distributional semantic spaces. Word embedding spaces show a crosslinguistically similar structure (Mikolov et al., 2013c), but even the most successful cross-language word embedding techniques learn the monolingual spaces separately, and only in a second step map the two languages onto each other (Artetxe et al., 2017, 2018; Lample et al., 2018). Other effective approaches have depended on sentence-aligned parallel corpora to support cross-lingual embeddings (e.g., Gouws et al., 2015; Levy et al., 2017; Zennaki et al., 2019, among others). These kinds of techniques may lead to multilingual spaces that show the influence of the particular resources and languages used. In short, there has been much work on multilingual approaches, but multilingual does not necessarily equal universal, which implies a common conceptual representation across languages. For example, one approach has required manually-specified conceptual categories to show improvements on both similar and distant languages (Wang et al., 2019). Further insights from linguistic and cognitive constraints on what is a valid lexical representation or a structured lexicon may further enable true crosslinguistic generalization.

In summary, computational approaches based on lexical and grammatical theories have developed richly structured lexicons, but achieving adaptability and crosslinguistic validity in such frameworks requires much manual effort. By inducing representations from data, current distributional semantic approaches have the potential to be fully adaptable, and generalizable across languages without the manual effort of earlier NLP systems. However, distributional research has largely focused on semantic similarity as the sole organizing principle of the learned knowledge, with less attention to the many other semantic relations encoded in the human lexicon. Moreover, despite their fundamental basis in learning, the proposed methods cannot adapt dynamically due to cognitively unrealistic training data requirements. Finally, although the learning methods are in principle generalizable across languages, they lack the biases to capture human conceptual underpinnings. As it stands, overcoming the weakness of the conceptual biases requires extremely large training data sets, available only for a few languages.

## 4. Inspiration From Human Lexical Abilities

Early work on lexical resources and automatic lexical acquisition had a strong basis in linguistic and psycholinguistic theory and insights. These connections have become more tenuous in recent NLP, despite earlier recognition that work on distributional representations in both CL and cognitive science can inform each other (see, e.g., Lenci, 2008), and despite continued work at the intersection of the two fields.^6^ A more concerted effort is required to bring linguistic and psycholinguistic understanding together with recent data-driven approaches in order to achieve more human-like lexical representations and abilities. Here we describe some relevant cognitively-inspired work from recent years, and suggest how such work can inform future directions in NLP to address the properties of the human lexicon.

### 4.1. Structure in Lexical Representations and Learning

Word embeddings are largely founded on the notion of semantic similarity, and ensuring that word vector similarities match human judgments has been an important goal (e.g., Baroni et al., 2014; Pereira et al., 2016; An et al., 2018; Grand et al., 2018; Iordan et al., 2022). Less attention has been paid to whether the actual structure of a DSM's similarity space matches what is known about the human lexicon. For example, while work in CL has noted that different types or levels of similarity may be captured in DSMs—first-order similarity reflecting word associations, and second-order similarity reflecting substitutability (e.g., Schütze and Pedersen, 1993; Grefenstette, 1994; Levy et al., 2015)—less attention has been paid to whether and how these finer-grained notions of similarity within current word embeddings match human lexical processing. Some recent work has addressed this issue (e.g., Beekhuizen et al., 2019; Chronis and Erk, 2020; Samir et al., 2020). For example, Samir et al. (2020) demonstrate that using different combinations of the input and output matrices of the word2vec algorithm not only mimics the two kinds of similarity, but does so in a way that matches human behavioral data on semantic priming and lexical decision. However, other properties of human similarity judgments—such as asymmetries in word associations or violations of the triangle inequality (*w*_1_ similar to *w*_2_, and *w*_2_ similar to *w*_3_, do not imply *w*_1_ similar to *w*3; cf. *asteroid, belt*, and *buckle*, Griffiths et al., 2007)—are not consistently captured in embedding spaces (Griffiths et al., 2007; Nematzadeh et al., 2017; Rodriguez and Merlo, 2020). Building on the insight from Griffiths et al. (2007) that interpretation of a word within the context of a topic can resolve some of these mismatches with human judgments by appropriately disambiguating the words, one avenue for the future may be to consider word embeddings that are topically-constrained (such as in Iordan et al., 2022).

Word embeddings also fail to reflect other linguistically-relevant types of similarity that play a role in human language processing. For example, when faced with long-distance dependencies between two feature-sharing items in a sentence (such as those found in questions, relative clauses, pronoun anaphora, and other frequent phenomena), people exhibit effects of interference if there is a third similar element in the sentence (Rizzi, 2004; Franck et al., 2015). However, this effect of similarity interference is not correlated to the similarity of words calculated statically in a vector space or even dynamically in a neural network model of processing (Merlo and Ackermann, 2018; Merlo, 2019). The general picture that emerges from all these studies is that word similarity is a rich construct of the human lexicon, and while word embedding spaces represent some fundamental properties of semantic similarity, more nuanced notions, and some grammatically-relevant aspects, may not emerge from such representations.

Moreover, work in psycholinguistics has shown that human access and interpretation of a word are influenced by its semantic neighborhood—the structural layout in semantic space of semantically similar words (e.g., Burgess, 1998; Buchanan et al., 2001). Such considerations are especially important for understanding how ambiguous words encode their varied semantics. Recent work has shown that some, but not all, oft-used word embeddings reflect a difference in neighborhood structure between homonyms (words with multiple unrelated meanings) and polysemes (words with multiple related senses) (Beekhuizen et al., 2018, 2021), corresponding to experimental differences in human processing of ambiguous words (Rodd et al., 2002; Hino et al., 2006). Other work has shown that contextualized (token) representations of abstract and concrete words differ in their pattern of dispersion across different layers of a language model (Chronis and Erk, 2020), again demonstrating the potential richness of lexical semantic structure. Such work illustrates that representational adequacy of distributional semantic models should consider finer-grained details than a match to human similarity judgments.

Beyond the similarity structure of word embeddings, it remains unclear how much current models capture the many other semantic relations that people are sensitive to (e.g., Köper et al., 2015; Santus et al., 2016; Ettinger, 2020). For example, the extent to which distributional semantic spaces represent more abstract semantic properties is an open question (Baroni and Lenci, 2008; Rubinstein et al., 2015; Hollis and Westbury, 2016; Hollis et al., 2017). Abstract semantic classes, such as verb or adjective classes, play a crucial role in theories of how human lexical knowledge encodes knowledge of grammar (e.g., Levin, 1993; Paradis, 2001; Morzycki, 2012), and so it is important that lexical representations support organization of such classes. For example, semantic verb classes are an important means for generalizing knowledge of argument structure: learning that a new verb *gorp* reflects a change of state will enable an English speaker to know that if you can say *Jane gorped the cookie*, you can also say *The cookie gorped*. Such classes often capture commonalities at a higher level of abstraction than the simple within-domain similarity that is typically demonstrated in word embeddings; for example, the change of state class covers verbs as dissimilar as *age, blacken, crumble, deflate*, and *energize* (Levin, 1993). Some recent work has demonstrated the ability of word embeddings to capture an abstract semantic class of adjectives that, like verb classes, also has ramifications for appropriate use of the words in grammatical constructions (Samir et al., 2021). However, even for the “poster child” task of solving linguistic analogies, which has showcased the semantic abilities of modern distributional representations (Mikolov et al., 2013b), higher levels of abstraction can be a challenge. It has been shown that for more abstract relations (ones that go beyond within-domain similarity) it is difficult to achieve reasonable performance in these tasks (Rogers et al., 2017), requiring more explicit knowledge of abstract classes (Drozd et al., 2016) or an additional learning component to extract the relevant dimensions of comparison (as in Lu et al., 2019).

Better understanding of learning algorithms may be required to achieve the kind of rich and abstract structure that human lexical knowledge demands. Again, insight may be drawn from cognitive principles. Analogies to human processes of memory and attention abound in neural architectures, yielding interesting and powerful mechanisms to guide the information flow through the network (e.g., Hochreiter and Schmidhuber, 1997; Vaswani et al., 2017). These mechanisms take inspiration from human cognition in an intuitive and loose sense, but generally do not distinguish the different types of mechanisms—such as working memory vs. episodic or semantic memory—found in humans. Closer modeling of the more structured findings from psychology and cognitive science might bring further fruits. For example, recent modeling of human reading processes in neural architectures has yielded finer-grained understanding of attention to words in language models (Sood et al., 2020; Hahn and Keller, 2021). Other work has noted that structured memory, as in humans, may be required for the kind of meaningful compression in learning that is necessary for successful abstraction over input stimuli: by disentangling computation and storage (which are intertwined in the weight parameters of most neural networks), richer storage mechanisms can be achieved that support both faster retrieval, and forgetting in support of abstraction (Nematzadeh et al., 2020). Other research considering cognitive factors in communication has found that human lexical organization is subject to optimization of the trade-off between complexity and accuracy (e.g., Kemp et al., 2018; Zaslavsky et al., 2018, among many others). The same principle of an information bottleneck has also been shown to help explain hierarchical structure in DNN layers (Tishby and Zaslavsky, 2015). Altogether, studies such as these suggest that drawing clearer connections between human principles of communication and current learning approaches may lead to more human-like lexical representations.

### 4.2. Adaptability and Learning From Small Amounts of Data

Adaptability requires generalization. But current data-driven NLP models do not generalize well to new problems or instances out of the training distribution (Ettinger et al., 2017; Belinkov and Bisk, 2018; Schölkopf, 2019). People are not as susceptible to overfitting, at least partly because they have strong prior biases, grounded in the actual causal structure of the problem. One possible approach for developing more robust methods, then, is to pay more attention to causal chains in the generative process that give rise to the data and not just to correlations in the data (Schölkopf et al., 2012; Lake et al., 2017; Bengio et al., 2020). Drawing on such causal knowledge should enable methods that support appropriate generalization, and improved adaptability.

Detailed linguistic analyses and psycholinguistic studies can provide information on the causal structure that is likely to underlie the observed distributions. For example, recent work in linguistics has investigated the causes of variation in the expression of causative constructions in several languages—corresponding to the alternation in English between *Kiva broke the vase* (with the causal agent specified) and *the vase broke* (Alexiadou, 2010; Haspelmath et al., 2014; Heidinger, 2015). A superficial correlation has been found between the distribution of verb form (length of the causative alternative of the verb) and its frequency (Haspelmath et al., 2014). However, further investigation has identified the perceived probability of external causation—is the event spontaneous or not—as a better explanation of the patterns of crosslinguistic data (Samardžić and Merlo, 2018). Taking this latent factor into account is shown to inform generalization, achieving improved prediction of which verbs can occur in which causative constructions (Samardžić and Merlo, 2018). In another example, Yu et al. (2020) proposed a probabilistic model building on linguistic analyses of denominalization—i.e., use of a noun as a verb (Clark and Clark, 1979). Human-like interpretation of novel uses—inferring that *porch the newspaper* likely means “throw the newspaper onto the porch”— depends on a latent frame (topic or scenario) variable in the model. Yu et al. (2020) demonstrate that this latent variable enables the model to outperform BERT in predicting the appropriate paraphrase for novel denominal verbs. This work suggests that appropriately modeling the causal structure of a phenomenon can outweigh even the massive knowledge encoded in a recent language model.

Such predictive generalization is key to achieving the on-the-fly adaptability that people exhibit. Historical corpora and other resources (including associated historical embeddings) have facilitated work on lexical change over some period of time (Hamilton et al., 2016; Lu et al., 2019), but less attention has been given to the rapid adaptation to novel nuances of meaning and novel constructions (e.g., Cook et al., 2014; Del Tredici et al., 2018; Ryskina et al., 2020; Watson et al., 2021). People continually produce words in new meanings and in creative usages of constructions, and interlocutors quickly extend their lexical knowledge to grasp the novel interpretations. This “one-shot” learning in people has not been achieved in recent NLP systems, which, as noted above, do not actually use just small amounts of training data in so-called “few-shot” learning (Perez et al., 2021). Psycholinguists have proposed a number of biases that enable children to learn words with few exposures; what principles govern the ability of adults to similarly adapt quickly and generalize over small amounts of data?

Much psycholinguistic work aims to elucidate the cognitive mechanisms that enable people to generalize their lexical knowledge in producing and interpreting novel usages of words. By understanding the cognitive processes at play when people form generalizations, work in NLP can better identify the factors and mechanisms required to achieve human-like abilities. For example, generalization of constructions to new words—such as saying “don't try to batman your way into it”—is viewed as a process of category extension (i.e., seeing a construction, such as “VERB one's way into NOUN,” as a category of usages). This process is influenced by factors such as similarity of the novel item to observed instances of the construction, and the frequency and variability of the latter—factors which support easier extension to new usages (e.g., Bybee and Eddington, 2006; Suttle and Goldberg, 2011; Perek, 2016). Recently, Watson et al. (2021) have demonstrated that these principles hold in creative usages in large-scale social media data – specifically, in novel usages of denominal verb constructions in an online discussion platform. For example, novel usages such as “I am a man (...usually all *flannelled up*)” tend to have high similarity to existing usages (*gear up, glove up, mask up, sweater up*, ...) that form a broad and frequent class. Moreover, Watson et al. (2021) find that novel usages cluster around other novel usages (*flannel* is similar to *sweater*), confirming that the exemplar-driven innovation found in historical analyses (e.g., Habibi et al., 2020; Yu et al., 2020) plays a role in dynamic adaptation of language. This is an important point, because one-off usages, rather than being statistical noise, serve as informative signals to people of legitimate creativity. Research is needed to see how such biases suggested by cognitive principles might be built into neural models of meaning acquisition and extension (e.g., compare McCoy et al., 2020), to ensure the level of lexical adaptability observed in human communication.

### 4.3. Truly Crosslinguistic Generalization

Semantic typology has contributed significantly to our understanding of the crosslinguistic foundations for human lexical semantics. Clearly, languages vary widely in how they “carve up” a semantic space with words—e.g., some having a single word for two concepts for which others have distinct words (English *on* [support] vs. Dutch *aan* [tenuous support] and *op* [stable support]). Despite this lack of alignment in the world's lexicons—with various one-to-many or even many-to-many mappings attested between languages—detailed linguistic analyses of various semantic domains have revealed consistent commonalities in how languages label concepts with words (e.g., Berlin and Kay, 1969; Haspelmath, 1993; Levinson et al., 2003; Majid et al., 2008; Gentner and Bowerman, 2009). More recently, large-scale work has confirmed that languages exhibit universal tendencies in lexical structure across a wide variety of semantic domains (Youn et al., 2016; Thompson et al., 2018). However, while NLP has effectively drawn on linguistic typology in other areas (such as morphology and syntax), little research has considered how to incorporate the insights from lexical semantic typology to inform and constrain computational approaches to meaning (e.g., Bender, 2016; Dubossarsky et al., 2019).

The creation of multilingual semantic spaces is one area that may benefit from typological considerations, especially an examination of how well such spaces capture the crosslinguistic principles that underlie human lexicons. Much of the richness of lexical structure, and differences across languages, arise from variation in polysemy—how languages differently package up related meanings into ambiguous words. For example, while the word for “tongue” in English, Hebrew, and Russian refers to both the physical organ and a language, only in English and Hebrew does it also refer to a piece of land that protrudes into the sea (Navigli and Ponzetto, 2010). Such misalignments pose serious challenges for NLP, since ambiguous words in one language can map very differently to words in another language. Rabinovich et al. (2020) showed that a multilingual semantic space could capture the similarity structure among concepts that match human patterns of such polysemies. Ensuring that multilingual spaces have such properties may enable them to better support automatic alignment across languages in future NLP systems.

One issue raised by such work is the extent to which multilingual spaces show bias from certain languages, since they generally rely on monolingual spaces or bilingual resources, in which some languages are likely over-represented (e.g., Artetxe et al., 2017, 2018; Lample et al., 2018). Interestingly, Merlo and Rodriguez (2019) show that multilingual spaces exhibit transfer effects—where the source language influences the semantic space of the target language—that are consistent with the cross-language influences seen in human bilinguals. This is intriguing, as it tells us that we can learn much from the broad literature on lexical semantic transfer effects in humans (e.g., Van Hell and de Groot, 1998; Degani et al., 2011). For example, even highly skilled human translators exhibit statistically detectable evidence of the source language in their target word choice (Rabinovich et al., 2017). By understanding more clearly how a source language can influence a target language, better means might be determined for anticipating bias in transfer learning and alleviating it. Such considerations are becoming increasingly important: work in NLP is heavily invested in so-called “foundation models,” which are largely focused on English due to their data and/or computation requirements (Bender et al., 2021; Bommasani et al., 2021). Methods for transferring such large-scale knowledge to a broad range of diverse languages will be necessary, and informed debiasing—drawing on knowledge of crosslinguistic tendencies and divergences—could be key to such efforts.

In addition to considering transfer between languages, NLP could also benefit from additional insights into the universal basis of lexical semantics. A key finding in semantic typology is the observation that, the more frequently (across languages) that two meanings are referred to by a single word, the more likely those meanings are to be (“universally”) semantically similar (e.g., Levinson et al., 2003; Gentner and Bowerman, 2009). Building on this insight, recent computational modeling work has shown that patterns in crosslinguistic data can reveal conceptual similarities that can form the basis of a “universal” semantic space for various lexical domains (Beekhuizen et al., 2014, 2017; Beekhuizen and Stevenson, 2018). In contrast to the typical multilingual approach in NLP of aligning a collection of monolingually-derived spaces, here a common semantic space across languages is founded in the dimensions of meaning that emerge from the crosslinguistic lexical patterns of aligned word usages. Such representations can reveal important properties of a distributional semantic space that conforms to typological principles. For example, building on insights from such a semantic space, Rabinovich et al. (2019) derived typologically-predicted patterns of human use of semantically-nuanced words, and demonstrated that some neural language models mimic these patterns. Practical limitations have prohibited the token-level, word-aligned techniques of Beekhuizen and colleagues from deployment for large-scale broad-coverage lexicons. However, there has been success in supersense tagging using coarser-grained type-level representations based on sentence alignments (Zennaki et al., 2019). Such results suggest that discovering similar methods for finer-grained representations that can scale is a promising avenue to pursue. Moreover, because their success depends on having a representative sample of languages, these kinds of approaches can inform how to sample languages efficiently to capture the broad crosslinguistic regularities (e.g., Stoll and Bickel, 2013; Beekhuizen and Stevenson, 2015; Beekhuizen et al., 2017). Thus, drawing on typological principles could extend the repertoire of NLP approaches to creation of multilingual spaces that truly generalize across languages.

## 5. Concluding Thoughts

The research from adjoining fields of linguistics, psycholinguistics, and cognitive science provides many challenging targets—as well as many sources of inspiration—for learning more structured, adaptable, and generalizable models in NLP. We have highlighted a broad range of interdisciplinary work that indicates how these high-level goals translate into more specific questions and hypotheses about computational approaches to word meaning. Such studies have informed the current understanding of human-like lexical representations and the algorithms that can achieve them, and have highlighted the possibilities for future research aimed at bringing these insights into NLP.

First, interdisciplinary research points to the need to learn more richly-structured notions of semantic similarity and other types of relations. Representations must achieve a higher level of abstraction that identifies classes of words that capture generalizable knowledge. The overarching challenge is for neural architectures to learn such structured semantic spaces. Research suggests that drawing closer connections to human cognitive mechanisms, such as memory, attention, and communicative efficiency, can lead to insight into what to store in memory and how to effectively abstract and simplify representations. Second, to achieve human levels of lexical adaptability, we must move beyond correlation to causation: systems must be sensitive to the latent causal factors of the observed effects, in order to support generalizations that mirror the structure of the problem, and thus are both more predictive and more explainable. To do so will require consideration of cognitive mechanisms such as categorization, and incorporation of human-like biases, such that learning systems can adapt dynamically given small amounts of data (even within a conversation). Finally, the goal of true crosslinguistic validity will require lexical representations that conform to a universal conceptual foundation, and multilingual semantic spaces that reflect the understood mappings between language-specific lexicons. Practical learning algorithms will need to anticipate transfer effects when using more-resourced languages to leverage knowledge for less-resourced languages. Multilingual systems will also need to draw on the known dimensions of typological, historical, and structural variation to inform small but representative language samples to ensure crosslinguistic generalization.

We have undergone a paradigm shift in natural language processing due to the ability of recent machine learning methods to effectively process huge amounts of data. But the integration of machine learning methods into computational linguistics is not new. The statistical revolution of the 1990s led to tremendous advances in a vast array of applications, from machine translation to automatic lexical acquisition to summarization and more. It also eventually led to a realization that knowledge of the language sciences—that is, deep understanding of the findings from fields like linguistics and psycholinguistics, on both the qualitative and the quantitative properties of language—were critical to obtaining success in NLP. Today, the same marvel of very large scale language models that is having such a positive effect on our ability to generate useful applications with relatively simple fine-tuning, has the negative effect of making us forget that grounded natural language processing is far from solved. Moreover, scientific progress is held back when resources and efforts are concentrated into the single mould of NLP as generic optimization, and away from questions and techniques that are more deeply integrated with the properties of the object of study. The language sciences have long (in some cases, thousands of years) revealed subtleties of the linguistic system that may be fruitfully incorporated into current approaches in NLP as knowledge representations, inductive biases, and principles of constrained variation.

## Author Contributions

PM and SS contributed equally to the ideas in and writing of this article and approved the submitted version.

## Funding

SS was supported by grant RGPIN-2017-06506 from NSERC (Canada). PM gratefully acknowledges the partial support of the NCCR Evolving Language, Swiss NSF Agreement 51NF40_180888.

## Conflict of Interest

The authors declare that the research was conducted in the absence of any commercial or financial relationships that could be construed as a potential conflict of interest.

## Publisher's Note

All claims expressed in this article are solely those of the authors and do not necessarily represent those of their affiliated organizations, or those of the publisher, the editors and the reviewers. Any product that may be evaluated in this article, or claim that may be made by its manufacturer, is not guaranteed or endorsed by the publisher.

## Footnotes

^1^In his LREC 2004 talk, “Some of my best friends are linguists,” Jelinek indicates the quote is from a talk entitled “Applying Information Theoretic Methods: Evaluation of Grammar Quality,” given at the Workshop on Evaluation of NLP Systems, Wayne PA, December 1988. The focus on statistical approaches as “the answer” in 1990s NLP is further illustrated by the loss of the context of this quip: while stressing that data-driven prediction models were key, Jelinek also emphasized the importance of input from linguistics on data annotation and model structure.

^2^Perhaps the quote that best sums up the current situation is “deep learning is going to be able to do everything” (Geoff Hinton, November 2020, when asked about the ability “to replicate all of human intelligence,” including language processing; https://www.technologyreview.com/2020/11/03/1011616/ai-godfather-geoffrey-hinton-deep-learning-will-do-everything/). For a recent informative debate on the tension—and potential for complementarity—between neural approaches and linguistic theory, see, for example, the perspective article of Pater (2019) and its associated commentaries.

^3^We are not alone in decrying the negative effects of the singular focus on (and sometimes exaggerated claims about) large language models, and associated benchmark tasks. See, for example, Church (2017), Bender and Koller (2020), and Church and Liberman (2021), and the carefully articulated recent discourse at https://twitter.com/emilymbender/status/1430944351358648324.

^4^While recognizing that the definition of “word” can be problematic and may have differing instantiations across languages, for simplicity here we will refer to lexical items as “words,” assuming this includes relevant morphemes, lexicalized multi-word expressions, etc.

^5^We fully recognize that semantic similarity and semantic relatedness are different constructs (e.g., Budanitsky and Hirst, 2006, among many others), and perhaps different as well from distributional similarity and relatedness (e.g., Sahlgren, 2008). Since the distinction between similarity and relatedness is not of high relevance to most points being made here, we will use “semantic similarity” to encompass the idea of closeness in semantic space, except where explicitly noted.

^6^See, e.g., the proceedings of regularly-occurring workshops such as CogACLL, CogALex, and CMCL, as well as tracks on linguistics and psycholinguistics in the major CL conferences.
